# Novel axial compressive endoprosthesis ACE can enhance metaphyseal fixation and facilitate osseointegration: A biomechanical study

**DOI:** 10.3389/fbioe.2022.1004849

**Published:** 2022-12-01

**Authors:** Siyi Huang, Tao Ji, Xiaodong Tang, Wei Guo

**Affiliations:** ^1^ Musculoskeletal Tumor Center, Peking University People’s Hospital, Beijing, China; ^2^ Key Laboratory for Musculoskeletal Tumor of Beijing, Beijing, China

**Keywords:** endoprosthetic reconstruction, metaphyseal fixation, osseointegration, segemental bone defect, diaphysis, compressive

## Abstract

**Background:** Metaphyseal fixation for endoprosthetic reconstruction after bone tumor resection is difficult due to the short residual bone length and reverse funnel shape of the metaphysis. In the current study, 3D-printed axial compressive endoprosthesis (ACE) with a short stem and intramedullary axial compressive mechanism is proposed to improve metaphyseal fixation. The rationales of ACE are that 1) intramedullary axial compress enhances the stability of endoprosthesis and facilitates bone ingrowth at the osteotomy site; 2) 3D printed porous metallic surface at osteotomy surface and stem allows bone ingrowth to achieve osseointegration.

**Methods:** A biomechanical study was performed to explore the initial stability using Sawbones. A diaphysis and metaphyseal segmental defect were created and four fixation structures were simulated: 1) ACE; 2) ACE + lateral plate; 3) stem prosthesis + unilateral plate; 4) stem prosthesis + bilateral plates. Bending and torsional stiffness were determined with a material testing machine. The relationship between the torque of the compression nut and the axial compression force of the bone-implant surface was measured using a round gasket load sensor.

**Results:** ACE + lateral plate was the stiffest in the bending test (sagittal 324.3 ± 110.8 N/mm, coronal 307.7 ± 8.7 N/mm). ACE + lateral plate and stem prosthesis + bilateral plates had the highest torsional stiffness (10.9 ± 1.3 Nm/° and 10.7 ± 0.2 Nm/° respectively). The bending stiffness of ACE was equivalent to stem prosthesis + bilateral plates (sagittal 196 ± 10 N/mm vs. 200 ± 7 N/mm, coronal 197 ± 14 N/mm vs. 209 ± 3 N/mm), but the torsional stiffness of ACE was inferior to stem prosthesis + bilateral plates (6.1 ± 1.3 Nm/° vs. 10.7 ± 0.2 Nm/°). Stem prosthesis + unilateral plate was the least stiff both in bending and torsion. The relationship between torque (T/Nm) and axial pressure (F/N) was F = 233.5T.

**Conclusion:** The axial compressive design of ACE enhances primary stability and facilitates osseointegration, which provides an alternative option of metaphyseal fixation for endoprosthetic reconstruction.

## 1 Introduction

Thanks to advances in imaging, chemotherapy and multidisciplinary treatment, the long-term survival of patients with primary malignant bone tumors has seen a significant increase in recent decades, and is now approaching 80% (SEER Cancer Statistics Review (CSR), 1975–2017). Limb-salvage surgery is now achievable in most extremity tumors (Ferrari et al., 2012). For malignant bone tumors involving metaphysis or diaphysis of long bones, joint-preserving surgery is a feasible choice (Panagopoulos et al., 2017). The reconstruction options are allografts, devitalized autografts, vascularized fibular grafts, or combined with allograft and segmental endoprosthesis (Panagopoulos et al., 2017).

Endoprosthetic reconstruction has the advantages of early weight-bearing, rapid rehabilitation, and satisfying function, however, the long-term survival is unsatisfactory with a high rate of structural failure and implant loosening (Ruggieri et al., 2011; Sewell et al., 2011; Benevenia et al., 2016). Metaphyseal fixation is frequently encountered at one or both ends of joint-preserving surgery, which is challenging due to the short remaining bone segment and reverse funnel shape of the metaphysis. The short remaining bone segment results in insufficient bone/cement or bone/implant interface (Qu et al., 2015), and the reverse funnel shape of metaphysis means the stem was surrounded by less solid cancellous bone (Coathup et al., 2000), which all lead to an increased risk of aseptic loosening.

A variety of attempts have been made to reduce the aseptic loosening rate and improve metaphyseal fixation stability. Extracortical plates (Cobb et al., 2005; Stevenson et al., 2017), cross-pin (Bernthal et al., 2019), and interlocking screws (Streitburger et al., 2020) are applied to enhance stability. A hydroxyapatite-coated collar is used in cemented stem fixation to prevent wear particles from entering the medullary cavity (Cobb et al., 2005; Stevenson et al., 2017). A 3D-printed stem with a porous structure is designed to achieve long-term osseointegration (Wang et al., 2021). However, there is still no well-established optimal technique for metaphyseal fixation.

A novel device 3D printed axial compressive endoprosthesis (ACE) with a short stem and the axial compressive mechanism is proposed by the senior author (Ji T) to improve metaphyseal fixation. The design rationales of ACE are that 1) intramedullary axial compression enhances initial stability of endoprosthesis and facilitates bone ingrowth at the bone-implant interface; 2) 3D printed porous metallic surface at osteotomy surface and stem allows bone ingrowth to achieve osseointegration.

In this study, metaphyseal fixation stability is compared between ACE and regular stem prostheses from an *in vitro* biomechanical test.

## 2 Materials and methods

### 2.1 Design and fabrication of ACE

**FIGURE 1:**
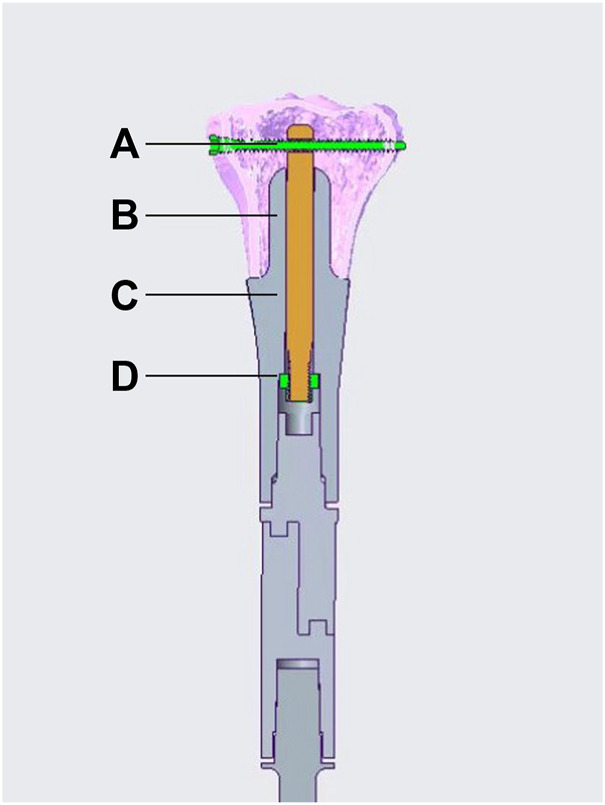
Schematic diagram of an ACE. **(A)** transverse interlocking screw, **(B)** axis rod, **(C)** stem, **(D)** compression nut. The set screw is not illustrated in the diagram.

**FIGURE 2:**
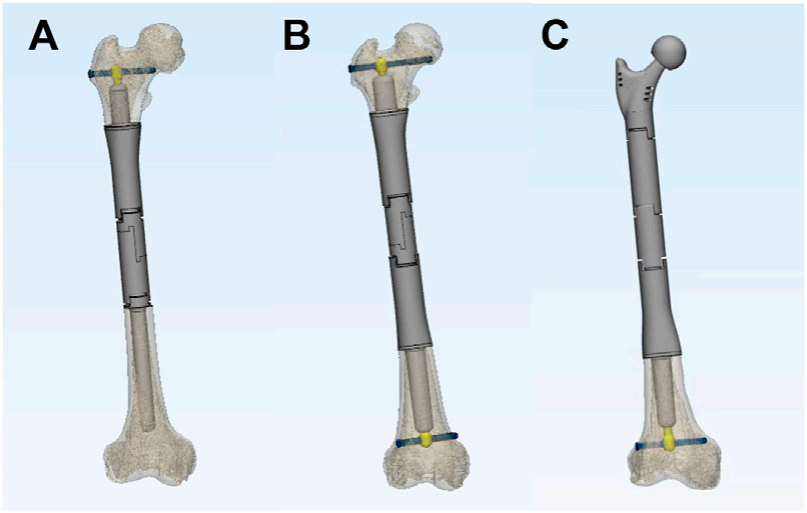
The versatility of ACE. ACE can be combined with a regular cement/press-fit stem **(A)**, double-ACE **(B)**, or an endoprosthesis with joint **(C)** for different ranges of bone defects.

The ACE is custom fabricated, based on the patients’ specific CT and MRI data. It is composed of five parts, stem, axis rod, transverse interlocking screw, compression nut, and set screw (Figure 1). The length and diameter of the stem are determined by the medullar geometry of the bone stump. The bone-contact surface of the stem is a layer of porous metallic structure created by 3D printing, allowing bone ingrowth. The other end of the stem is a taper structure that can be connected with regular modular megaprostheses. It can be assembled into different types on the other end providing flexible reconstruction solutions, a regular cement/press-fit stem, another ACE, or an endoprosthesis with a joint based on the length of residual bone on the other end (Figure 2). The stem of ACE is hollow and an axis rod (diameter, 8–10 mm) passes through the center of the stem. A 6.5-mm transverse interlocking screw is placed through the end of the axis rod within the metaphyseal bone. The other end of the axis rod is threaded with a compression nut. When the compression nut is tightened, the interface between ACE and bone is compressed and the ACE is fixed to the bone stump. There is a groove on the axis rod. The set screw can be fixed on the groove through the endoprosthesis to prevent the axis rod from rotating relative to the stem. A lateral plate can be screw fixed to the prosthesis to provide additional stability, mainly for anti-rotation purposes. The ACE is fabricated by 3D printing with Ti6Al4V alloy using the electron beam melting technique (ARCAM Q10plus, Molndal, Sweden).

### 2.2 Biomechanical testing

#### 2.2.1 Study groups and instrumentation

Fourth-generation bone composite models (Sawbones, Pacific Research Laboratories, Vashon, WA, United States) were selected instead of cadaveric bones as composite models simulate accurately the biomechanical behavior of human bones (Gardner et al., 2010; Elfar et al., 2014), avoiding the disadvantages of variety in geometry, strength and bone density. In total, 25 adult femur specimens (#3403–105) were used for the biomechanical study.

**FIGURE 3:**
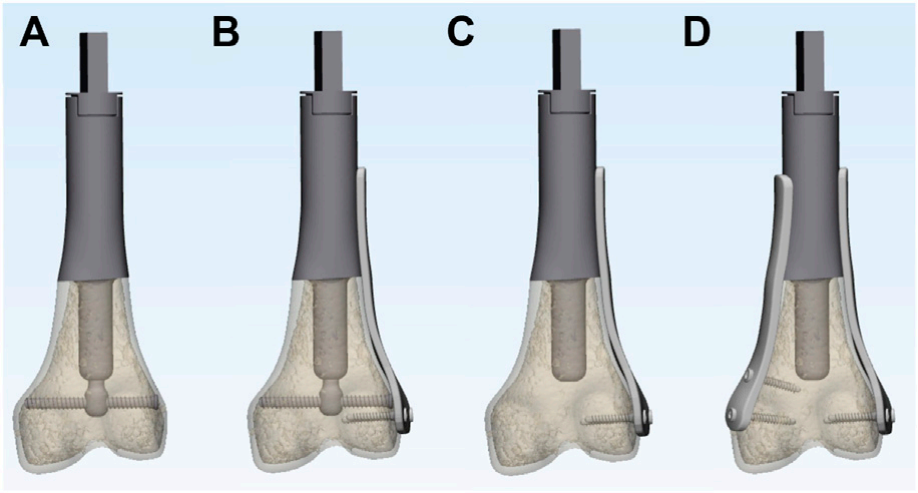
3D models of the 4 different experimental reconstruction groups: **(A)** ACE; **(B)** ACE + lateral plate; **(C)** stem prosthesis + unilateral plate; **(D)** stem prosthesis + bilateral plates.

To simulate typical metaphyseal fixation, all composite models of the femur were marked and cut to create identical defects. The osteotomy was planned at 9 cm from the joint surface of the femoral condyle and perpendicular to the long axis of the femur. The bone defects were then reconstructed using four different options: 1) ACE; 2) ACE + unilateral plate; 3) stem prosthesis + unilateral plate; 4) stem prosthesis + bilateral plates (Figure 3). Each group had 6 specimens. The stem of ACE in this study was 5 cm in length and 17 mm in diameter. The surface of the stem featured a layer of metallic porous structure with 2 mm thickness. The compression nut was tightened to 5 Nm for compressive fixation. The same stem of ACE was used for the stem prosthesis reconstruction, and the compressive structure was removed. The medial and lateral plates were fixed to the prosthesis. Each plate stabilized the bone with two locking screws.

#### 2.2.2 Stiffness testing

**FIGURE 4:**
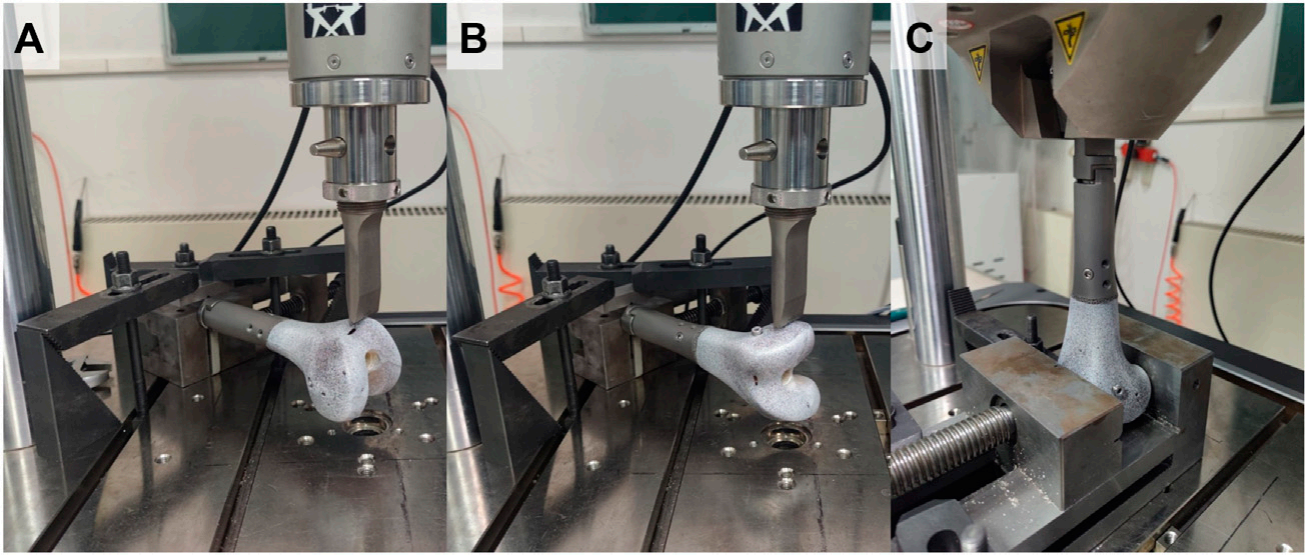
Test set-up: **(A)** sagittal bending test, **(B)** coronal bending test, **(C)** torsional test.

An Instron E10000 materials testing machine (Instron, Canton, MA, United States) was used to conduct the testing. Bending stiffness was determined in the sagittal plane (anterior cortex in tension) and coronal plane (lateral cortex in tension). The femur was held horizontally and the proximal end of the endoprosthesis was fixed in a vice (Figure 4). A load was applied to the apex of the anterior aspect of the femoral condyle and the lateral aspect of the femoral condyle for sagittal and coronal plane stiffness test, respectively. Mechanical loading was applied using a displacement control mode. A preload of 50 N was applied to stabilize the construct, calibrated, and then followed by 5 load cycles (Sakellariou et al., 2012). The data of the last three cycles were collected for analysis. The maximum displacement was set as 1 mm. The goal was to perform non-destructive testing and ensure that the specimens remained in the linear elastic portion of the load-displacement curve (Talbot et al., 2008). Load (N) versus displacement (mm) curves were generated directly from the load cell in the actuator. The slope of the ascending linear portion was used to compute the bending stiffness (N/mm) by linear regression.

In a torsional test, the construct was secured vertically with two ends (Figure 4). The rotation axis was aligned with the long axis of the construct. A preload of 1 Nm was applied to stabilize the construct, zeroed, and then followed by 5 load cycles. The data from the last three cycles were collected for analysis. One degree of torsion was set as a limit for non-destructive testing. Load (Nm) versus angle (degrees) curves were generated directly from the load cell in the actuator. The slope of the ascending linear portion was used to calculate the torsional stiffnesses (Nm/degree).

The loading was sequential following the same pattern for all specimens that were tested; the composite sawbones models were tested first for sagittal bending, then for coronal bending, and finally for torsion. After each cycle of loading the specimens were examined for implant loosening, the presence of cracks as well as the need for additional tightened of the screws.

#### 2.2.3 Torque—Axial compression force relationship test

The relationship between axis torque and axial compression force at the interface of bone-implant was measured using a gasket load sensor (JHBM-4-DZQ-500KG, Bengbu Jinnuo, Anhui, China). An ACE with a 6 cm-length and 20 mm-diameter stems was manufactured to allow the load sensor assembled. The torque of the compression nut was measured using a torque wrench (WWM-10, Wenzhou Weidu Electronics, Zhejiang, China). During the compression process, compression force (N) versus torque (Nm) was recorded. The experiment was repeated 6 times. Linear regression was used to calculate the relationship between the torque of the compression nut and axial compression force.

### 2.3 Statistical analysis

Statistical analyses were performed with SPSS software (version 20.0, Chicago, IL, United States). One-way analysis of variance (ANOVA) was used to test for any significant differences between the mean values of sagittal bending stiffness, coronal bending stiffness, and torsional stiffness of four different reconstructions. Levene’s test was used to assess variance homogeneity. If there was no significant difference between the variances of these sets of data, a post hoc Tukey test was used, or a post hoc Tamhane’s T2 test was used. The threshold for statistical significance was set at *p* = 0.05.

## 3 Results

### 3.1 Stiffness test

All specimens were loaded within linear elastic region at subyield level. There were no events of implant or bone composite model failures observed. No implant or screw loosening was detected after each mode of loading. No tightening of screws was needed.

The mean stiffness and standard deviation (SD) for sagittal bending, coronal bending, and torsion for each reconstruction are listed in Table 1. One-way analysis of variance (ANOVA) showed a statistically significant difference in each set of data. Specifically, the F ratio of sagittal bending stiffness was 336.8 (*p* < 0.001), of coronal bending stiffness 273.0 (*p* < 0.001), and of torsional stiffness 134.7 (*p* < 0.001). Levene’s test showed that the variances for sagittal bending stiffness and coronal bending stiffness were equal (*p* = 0.682 and *p* = 0.058, respectively), while the variances for torsional stiffness were not equal (*p* = 0.001).

**TABLE 1:** The mean stiffness and standard deviation for sagittal bending, coronal bending, and torsion for each reconstruction.

	ACE		ACE + lateral plate		stem prosthesis + unilateral plate		stem prosthesis + bilateral plates	
	Mean	SD	Mean	SD	Mean	SD	Mean	SD
sagittal bending (N/mm)	196.3	10.2	324.3	10.8	164.1	9.3	199.6	7.1
coronal bending (N/mm)	197.4	13.7	307.7	8.7	179.7	4.1	209.3	3.1
axial torsion (Nm/Degree)	6.13	1.28	10.94	1.28	1.78	0.13	10.67	0.21

**FIGURE 5:**
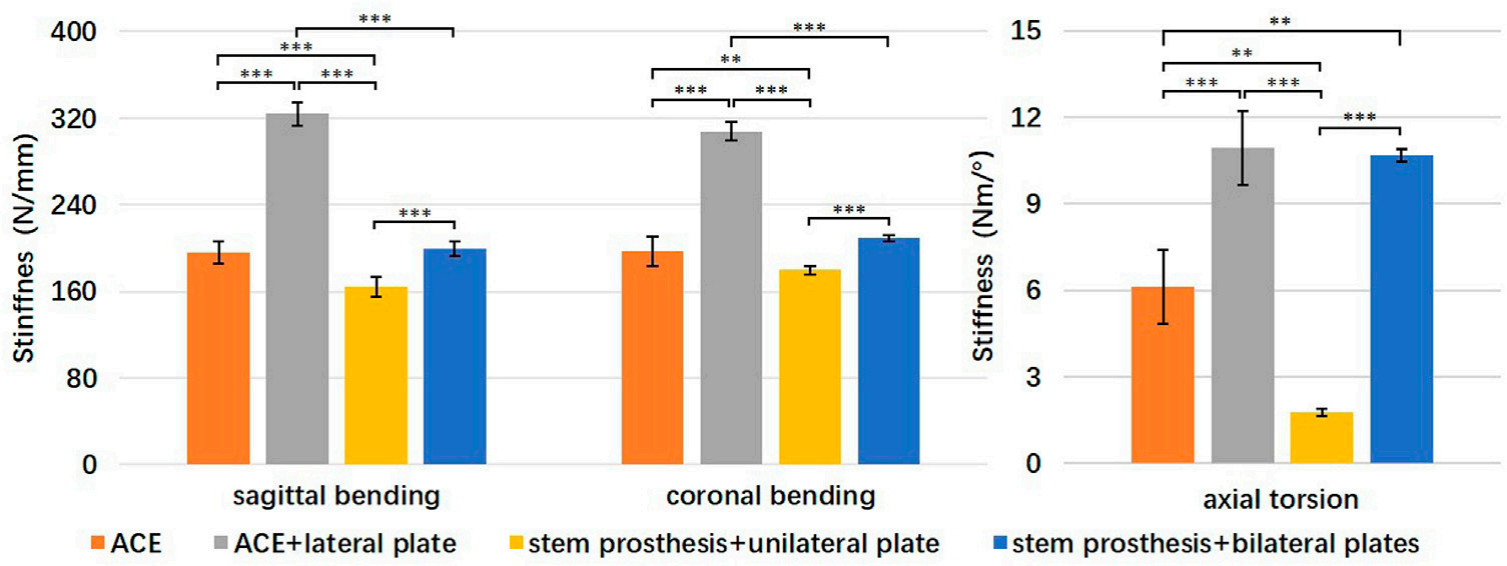
Comparison of bending and torsional stiffness of four reconstruction groups. ^**^ *p* < 0.01, ^***^ *p* < 0.001.

A post hoc comparison of groups was performed in sagittal bending stiffness and coronal bending stiffness using the Tukey multiple comparison procedure. Sagittal and coronal bending stiffness were statistically significantly higher for the ACE + lateral plate group compared to the other three groups (*p* < 0.001) (Figure 5). Sagittal and coronal bending stiffness were statistically significantly higher for the ACE and stem prosthesis + bilateral plates comparing to the stem prosthesis + unilateral plate (*p* < 0.01 in coronal bending, ACE versus stem prosthesis + unilateral plate, *p* < 0.001 in other three comparisons). No significant differences were found between ACE and stem prosthesis + bilateral plate in sagittal or coronal bending stiffness.

A post hoc comparison of groups was performed in torsional stiffness using Tamhane’s T2 test. Torsional stiffness was statistically significantly higher for the ACE + lateral plate and stem prosthesis + bilateral plates compared to the ACE and stem prosthesis + unilateral plate (*p* < 0.01 in ACE versus stem prosthesis + bilateral plate, *p* < 0.001 in the other three comparisons). Torsional stiffness was statistically significantly higher for the ACE compared to the stem prosthesis + unilateral plate (*p* < 0.01). No significant differences were found between ACE + lateral plate and stem prosthesis + bilateral plates in torsional stiffness.

### 3.2 Axial compression force–Torque relationship test

**FIGURE 6:**
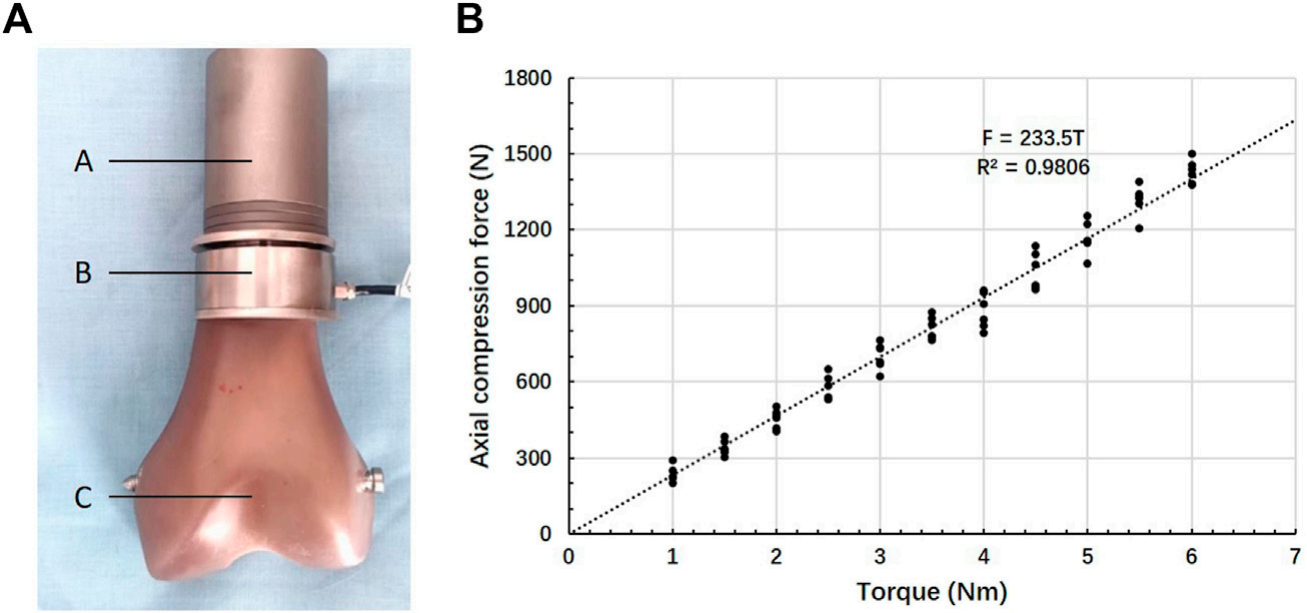
Compression force–Torque relationship. **(A)** Test set-up: A- ACE, B- gasket load sensor, C- Sawbones, **(B)** Relationship between torque and axial force.

The fitted regression equation of torque (T/Nm) and axial pressure (F/N) was as follows: F = 233.5T (Figure 6). The Pearson correlation coefficient was 0.9806. The axially compressive force of the bone-implant surface was 1,167.5 N when the torque of the compression nut was 5 Nm.

## 4 Discussion

Metaphyseal fixation of endoprosthesis reconstruction after segmental bone removal is usually difficult due to the short remaining bone segment and reverse funnel shape of the metaphysis. Traditional stems rely on a certain length of the stem being placed into a tubular bone for fixation. The reduced bone/cement or bone/implant interface (Qu et al., 2015) and relatively weak cancellous bone (Coathup et al., 2000) at metaphysis make both cemented stem and press-fit stem under an increased risk of aseptic loosening.

### 4.1 Efforts to improve metaphysis fixation

Efforts to improve metaphysis fixation can be summarized into two categories. One is auxiliary fixation structure to enhance reconstruction, the other is a surface modification to achieve osseointegration (Bini et al., 2000; Dieckmann et al., 2014; Stevenson et al., 2017; Bernthal et al., 2019; Wang et al., 2021).

Stevenson et al. (2017) reported that HA (hydroxyapatite) coated extra-cortical plates increased the survivorship of short-cemented-stemmed endoprostheses. In their study, the average length of the intramedullary stem was 33 mm. The follow-up results of 37 patients showed that no implants with extra-cortical plate osseointegration suffered loosening at a mean of 8.5 years, while three of ten (30%) without osseointegration suffered aseptic loosening at a mean of 7.7 years. Bernthal et al. (2019) reported the long-term outcomes of a cross-pin fixation construct designed to minimize rotational stress and subsequent aseptic loosening. The median follow-up of 56 implants was 132 months. Five implants (9%) were revised for aseptic loosening. However, three implants had a fatigue fracture of the stem through cross-pin holes in the endoprostheses. Dieckmann et al. (2014) reported a short-stem reconstruction with screw fixation in the femoral neck to avoid rotation and enhance primary rotation stability. Two aseptic loosenings were noted in a total of 15 patients during the mean follow-up period of 37 months. Wang et al. (2021) reported a 3D-printed custom-made ultra-short stem with a porous structure for metaphysis fixation. Cross-pins were used to enhance stability. One of 15 patients experienced aseptic loosening which was managed with immobilization and bisphosphonates infusion at a median follow-up of 42 months. All implants were well osseointegrated at the final follow-up.

The Compress prosthesis offered a new coupling system. It has a porous–coated titanium surface with a conical section mounted transversely to the axis of the bone. The implant is compressed against the host bone with Belleville spring washers tightened by a bolt over an intramedullary traction bar (Bini et al., 2000). The aim of the Compress prosthesis was to prevent bone resorption by allowing stress sharing by the implant and the bone, and provide a stable bone-prosthesis interface suitable for early weight bearing and long-term osseointegration. The aseptic loosening rate was 9.7% at 10 years (Healey et al., 2013). The conical spindle rather than an intramedullary stem was used in the Compress prosthesis. A compression force from 400 lbs to 800 lbs was applied to the osteotomy surface according to the cortical thickness (Parlee et al., 2020). A cortical thickness of less than 2.5 mm was a contraindication of this device. The Compress prosthesis required approximately 8 cm of resected bone at the distal femur, 13 cm at the proximal femur, 15 cm at the proximal tibia, and 12 cm at the proximal humerus (Parlee et al., 2020). The requirement of cortical thickness and resected bone length limited its application in metaphyseal fixation.

### 4.2 Superior stability of ACE

In the biomechanical testing, the ACE reconstruction had higher bending stiffness than the stem prosthesis + unilateral plate. The ACE + lateral plate had the highest bending stiffness among the four reconstructions. The interface between ACE and bone was compressed, which not only increased maximum static friction between bone and metal prosthesis but also prevented the pulling out of the tension side under bending force. The traditional stem relies on the press fit technique to provide friction, but the reverse funnel shape of metaphysis makes it not able to achieve press fitting.

The ACE reconstruction was superior to the stem prosthesis + unilateral plate on torsional stiffness while was inferior to the stem prosthesis + bilateral plate. The ACE + unilateral plate had similar torsional stiffness with the stem prosthesis + bilateral plate. The compression between ACE and bone increased friction. The transverse interlocking screw also enhanced the torsional stiffness. However, the screw hole on the axis rod was not threaded. There was a slight relative rotation between the axis rod and the transverse interlocking screw under torsion.

The above findings indicated that the intramedullary axial compress system was more stable than traditional stem plus plate fixation. The lateral plate fixed to the ACE provided additional initial stability, especially torsional stability.

The torque of the compression nut is set to 4 Nm and 5 Nm during surgery depending on the cortical thickness and quality of cancellous bone. The corresponding axial compressive force is 934 N and 1,167.5 N according to our test. The compressing force is from 400lbs (1778 N) to 800lbs (3555 N) in the Compress prosthesis. It is reasonable that the compression force is lower in the ACE than the Compress prosthesis, considering the thinner cortical bone at metaphysis than diaphysis.

### 4.3 Limitations

The present study had several limitations. Sawbones were used in the biomechanical experiments, which reflected the biomechanical properties of the early postoperative period, but not after bone ingrowth. Future research could carry animal studies to further investigate the effect of osseointegration on the mechanical properties of metaphyseal fixation. Only distal femoral metaphyseal fixation was evaluated in biomechanical experiments. We believe that the results can be applied to other long bone metaphyses, but further experimental verification is needed.

### 4.4 Conclusion

The axial compressive design of ACE enhances primary stability and has facilitated osseointegration, which provides an alternative option of metaphyseal fixation for endoprosthetic reconstruction.

## Data Availability

The raw data supporting the conclusion of this article will be made available by the authors, without undue reservation.
